# The majority of autosomal recessive nanophthalmos and posterior microphthalmia can be attributed to biallelic sequence and structural variants in *MFRP* and *PRSS56*

**DOI:** 10.1038/s41598-019-57338-2

**Published:** 2020-01-28

**Authors:** Basamat Almoallem, Gavin Arno, Julie De Zaeytijd, Hannah Verdin, Irina Balikova, Ingele Casteels, Thomy de Ravel, Sarah Hull, Martina Suzani, Anne Destrée, Michelle Peng, Denise Williams, John R. Ainsworth, Andrew R. Webster, Bart P. Leroy, Anthony T. Moore, Elfride De Baere

**Affiliations:** 1 Center for Medical Genetics, Ghent University and Ghent University Hospital, Ghent, Belgium; 2 Department of Ophthalmology, King Abdul-Aziz University Hospital, College of Medicine, King Saud University, Riyadh, Saudi Arabia; 3 Institute of Ophthalmology, University College London, London, United Kingdom; 4 Department of Ophthalmology, Moorfields Eye Hospital, London, United Kingdom; 5 Department of Ophthalmology, Ghent University and Ghent University Hospital, Ghent, Belgium; 6 Department of Ophthalmology, University Hospitals Leuven, Leuven, Belgium; 7 Center for Human Genetics, University Hospitals Leuven, Leuven, Belgium; 8 Department of Human Genetics, Institut de Pathologie et de Génétique (IPG), Charleroi, Belgium; 9 Department of Ophthalmology, University of California, San Francisco, California United States; 10 Birmingham Women’s NHS Foundation Trust, Birmingham, UK; 11 Division of Ophthalmology, The Children’s Hospital of Philadelphia, Philadelphia, Pennsylvania United States

**Keywords:** Hereditary eye disease, Molecular medicine

## Abstract

This study aimed to genetically and clinically characterize a unique cohort of 25 individuals from 21 unrelated families with autosomal recessive nanophthalmos (NNO) and posterior microphthalmia (MCOP) from different ethnicities. An ophthalmological assessment in all families was followed by targeted *MFRP* and *PRSS56* testing in 20 families and whole-genome sequencing in one family. Three families underwent homozygosity mapping using SNP arrays. Eight distinct *MFRP* mutations were found in 10/21 families (47.6%), five of which are novel including a deletion spanning the 5′ untranslated region and the first coding part of exon 1. Most cases harbored homozygous mutations (8/10), while a compound heterozygous and a monoallelic genotype were identified in the remaining ones (2/10). Six distinct *PRSS56* mutations were found in 9/21 (42.9%) families, three of which are novel. Similarly, homozygous mutations were found in all but one, leaving 2/21 families (9.5%) without a molecular diagnosis. Clinically, all patients had reduced visual acuity, hyperopia, short axial length and crowded optic discs. Retinitis pigmentosa was observed in 5/10 (50%) of the *MFRP* group, papillomacular folds in 12/19 (63.2%) of MCOP and in 3/6 (50%) of NNO cases. A considerable phenotypic variability was observed, with no clear genotype-phenotype correlations. Overall, our study represents the largest NNO and MCOP cohort reported to date and provides a genetic diagnosis in 19/21 families (90.5%), including the first *MFRP* genomic rearrangement, offering opportunities for gene-based therapies in *MFRP*-associated disease. Finally, our study underscores the importance of sequence and copy number analysis of the *MFRP* and *PRSS56* genes in MCOP and NNO.

## Introduction

Congenital microphthalmia (MCO) is a heterogeneous developmental eye disease characterized by small, hyperopic eyes secondary to several etiological factors affecting the early formation of the optic cup^1–3^. MCO has a reported incidence of approximately 15/100,000 live births per year^4^ and may be isolated, complex or syndromic based on the presence or absence of associated ocular malformations and systemic involvement^2,5^. Moreover, MCO can be subclassified into two clinical subtypes called nanophthalmos (NNO) and posterior microphthalmia (MCOP), based on the involvement (NNO) or not (MCOP) of the anterior segment^6,7^. Both NNO and MCOP may be associated with retinitis pigmentosa (RP), foveoschisis and optic disc drusen^8–10^.

Clinically both conditions are characterized by a short axial length that gives rise to high hyperopia ranging between +8.00 to +25.00 diopters^11^. Best-corrected visual acuity (BCVA) is reduced and rarely better than 20/40^12–15^. This poor BCVA is primarily caused by the hyperopia but may be aggravated by posterior segment changes such as an abnormal foveal structure and so-called papillomacular folds, uveal effusion, and an abnormal foveal avascular zone^13,16^. Patients with NNO or MCOP are prone to complications such as angle closure glaucoma, uveal effusion after intra-ocular surgery, non-rhegmatogenous retinal detachment and intraretinal cysts^13,16–18^.

Biallelic mutations in the *MFRP* (encoding membrane-type frizzled related protein, MIM 606227) or *PRSS56* (encoding protease serine 56, MIM 613858) genes have been reported to cause autosomal recessive NNO or MCOP. *MFRP*^19,20^ was found to be expressed predominantly in the retinal pigment epithelium (RPE) and ciliary epithelium of the eye, with a weak expression in fetal brain^3^. Mouse and zebrafish studies confirmed its expression in the RPE and ciliary body^20^. Zebrafish models recapitulated reduced axial length causing hyperopia, reduced visual acuity and RPE folding^21^. Apart from its role in eye development and emmetropization, MFRP plays a role in photoreceptor maintenance, explaining the risk for RP-like changes when mutated^8,22,23^. *PRSS56* was found to be expressed in human neural retina, cornea, sclera, and the optic nerve. Mouse *Prss56* is expressed in the eye from embryonic development until adult life^17^. Mouse studies and genome-wide association studies suggest that *PRSS56* is taking part of a regulatory network influencing postnatal eye development and emmetropization^24–26^.

Here, we aimed to genetically and clinically characterize 25 individuals from 21 unrelated families with autosomal recessive NNO or MCOP from different ethnicities, representing the largest cohort reported to date. This revealed eight distinct *MFRP* and six distinct *PRSS56* mutations respectively, providing a molecular diagnosis in 19/21 families (90.5%) and uncovering opportunities for gene-based therapies in *MFRP*-associated disease.

## Methods

### Patients and clinical assessment

Twenty-five patients out of 21 unrelated families from different ethnicities with either isolated or complex NNO or MCOP were recruited from three centers: Ghent University Hospital, Belgium (n = 11); Moorfields Eye Hospital, London, UK (n = 7); and Birmingham Children’s Hospital, Birmingham, UK (n = 7) (Table 1). A full ophthalmological examination included BCVA measurement and dilated fundus examination. Retinal fundus imaging was obtained by conventional 30-degree fundus colour photographs (Topcon Great Britain Ltd, Berkshire, UK) or via ultra-wide field confocal scanning laser imaging (Optos plc, Dunfermline, UK), near-infrared and blue light fundus autofluorescence (FAF) imaging (Spectralis, Heidelberg Engineering Ltd, Heidelberg, Germany), and spectral domain optical coherence tomography (OCT) scans (Spectralis, Heidelberg Engineering Ltd, Heidelberg, Germany). Full-field electroretinography (ERG) was performed according to the International Society for Clinical Electrophysiology of Vision (ISCEV) standards^27^ (using either Roland Consult, Brandenburg an den Havel, Germany or Diagnosys, Cambridge, UK equipment). Genomic DNA was extracted from EDTA blood using standard procedures. This study was conducted following the tenets of the Declaration of Helsinki.

**Table 1:** Clinical features of patients from 21 unrelated families with isolated or complex NNO or MCOP.

FA#	PT#	Extended ID	Sex	Ethnicity/Consanguinity	Age (year)	Group	Gene	BCVA, logMAR (Snellen)		Posterior segment features					AL (mm)		Posterior coat thickness (mm)		Refraction (diopter)	ERG	Diagnosis
								R	L	Crow-ded discs	Papillo-macular fold	Macular edema	White dots	Perip-heral pigment	R	L	R	L			
F1	P1	B03271	F	Belgian/Consanguineous	23	Ghent	*MFRP*	0.6	0.3	+	+	+	+	+	16.1	15.7	NA	NA	R + 14,00 /−0,50 × 13 L + 15,50 /−0,25 × 117	SN	NNO/RP
F2	P2	B14785	M	Moroccan/Consanguineous	3	Ghent	*MFRP*	0.4	0.5	+	+	-	+	-	14.5	14.4	NA	NA	R+12.00 / + 1.00 × 180 L + 12.00/ + 1.00 × 180	SN	MCOP
F3	P3	GC19623	F	Indian/NA	29	London	*MFRP*	0.5	0.5	+	-	+	-	-	13.3	12.8	2.1	2.1	R + 15.25/−0.25 × 175 L + 15.25/−0.50 × 155	NL	MCOP
F4	P4	GC19691	F	British Caucasian/NA	15	London	*MFRP*	0.2	0.3	+	+	-	+	+	15.5	15.5	2.1	2.1	R + 16.00/−0.5 × 50 L + 16.00/−0.5 × 100	SN	MCOP
F5	P5	B09352	F	Dagestanian/Consanguineous	68	Ghent	*MFRP*	0.3	0.1	+	-	+	+	+	16.5	16.5	NA	NA	R + 8,50 /−1,00 × 106 L + 8,75 /−0,75 × 113	NR	NNO/RP
F6	P6	GC18886	M	Kurdish/NA	31	London	*MFRP*	0.8	0.6	+	-	+	-	+	16.5	16.3	1.7	1.9	R + 17.5/−0.50 × 95 L + 16.00/−0.50 × 90	NA	MCOP/RP
F7	P7	GC20271	F	Iranian/NA	51	London	*MFRP*	0.6	1.3	+	-	+	-	+	NA	NA	NA	NA	R + 15.0/−1.00 × 100 L + 15.5/−0.75 × 110	SN	MCOP/RP
F8	P8	B10315	M	Italian/NA	26	Ghent	*MFRP*	LP	LP	+	-	+	+	-	15.7	15.7	NA	NA	R +15.25/−0.25 × 175 L + 15.25/−0.50 × 155	SN	NNO/RP
F9	P9	B16796	F	Belgian/NA	75	Ghent	*MFRP*	0.1	0.1	+	-	+	+	+	NA	NA	NA	NA	R+17,00/ 0,75 × 15 L + 15,00/ −0,75 × 170	SN	MCOP
F10	P10	B08047	M	Moroccan/Consanguineous	8	Ghent	*MFRP*	0.25	0.25	+	-	NA	NA	NA	NA	NA	NA	NA	R + 13.75 L+13.25	NA	NNO
F11	P11	GC20258	F	British Caucasian/NA	18	London	*PRSS56*	0.6	0.3	+	-	-	+	-	15.7	15.8	2.3	2.1	R + 15.00/−0.5 × 180 L + 13.50/−0.75 × 180	NL	MCOP
F12	P12	GC18588	M	Pakistani/NA	37	London	*PRSS56*	0.5	0.6	+	+	+	+	-	15.4	15.4	1.9	1.9	R + 2/−1.25 × 180 L + 4.5/−1.5 × 180	SN	MCOP
F13	P13	GC16899	M	Somalian/NA	20	London	*PRSS56*	0.6	0.6	+	+	+	+	-	16.5	16.4	1.9	2.1	R + 15.00/−0.75 × 110 L + 15.50/−0.50 × 75	SN	MCOP
F14	P14	GC19721	M	Pakistani/NA	23	London	*PRSS56*	0.8	0.8	+	+	-	+	-	15.9	15.7	2.1	2.1	R + 9.50/−1.00 × 20 L + 11.00/−1.00 × 40	SN	MCOP
F15	P15	RXK-4624916	F	Pakistani/Consanguineous	44	Birmin-gham	*PRSS56*	NA	NA	+	+	-	-	+	17	17	2.4	2.4	R + 15.00 L + 15.50	NA	MCOP/RP
	P16	RXK-3297132	M	Pakistani/Consanguineous	13	Birmin-gham	*PRSS56*	0.2	0.1	+	+	-	+	+	15.2	15.6	NA	NA	R + 14.00 L + 14.50	NL	MCOP/RP
	P17	RXK-33447157	F	Pakistani/Consanguineous	8	Birmin-gham	*PRSS56*	0.6	0.6	+	-	-	+	-	14.0	14.2	NA	NA	R + 16.00 L + 18.50/−1.00 × 10	SN	MCOP
	P18	RXK-3113374	F	Pakistani/Consanguineous	12	Birmin-gham	*PRSS56*	0.5	0.5	+	+	-	-	-	14.8	14.6	NA	NA	R + 15.50/−1.50 × 30 L + 14.50/−1.00 × 8	SN	MCOP
	P19	RXK-4633376	F	Pakistani/Consanguineous	17	Birmin-gham	*PRSS56*	0.5	0.5	+	-	-	-	-	14.5	14.5	NA	NA	R + 16.00/−1.00 × 180 L + 15.00/−1.00 × 180	NL	MCOP
F16	P20	RXK-4941281	M	Mirpuri/Consanguineous	7	Birmin-gham	*PRSS56*	0.5	0.1	+	+	-	-	-	16.0	16.2	2.4	2.4	R + 16.25/−1.00 × 180 L + 17.25/−1.75 × 180	NA	MCOP
F17	P21	RXK-4985235	F	Mirpuri/Consanguineous	10	Birmin-gham	*PRSS56*	0.5	0.5	+	+	-	-	-	15.0	15.1	2.8	2.7	R + 19.50 L + 19.50	NA	MCOP
F18	P22	B05802	M	Turkish/Consanguineous	4	Ghent	*PRSS56*	0.3	0.3	+	+	+	-	-	15.3	15.1	NA	NA	R + 14,25/ −0,50 × 4 L + 15,00 /−0,50 × 174	NL	MCOP
F19	P23	B01921	M	Bulgarian/NA	26	Ghent	*PRSS56*	0.4	0.5	+	+	+	-	-	14.8	14.6	NA	NA	R + 15.25/−0.25 × 175 L + 15.25/−0.50 × 155	SN	NNO
F20	P24	B03421	M	Belgian/NA	7	Ghent	/	0.3	0.4	+	+	+	-	-	14.5	14.5	NA	NA	R + 14,25 /−1.00 × 13 L + 15,25 /−1.75 × 173	NL	MCOP
F21	P25	B07457	M	Belgian/NA	8	Ghent	/	0.2	0.2	+	+	NA	NA	NA	NA	NA	NA	NA	R NA L NA	NA	NNO

Abbreviations used: FA#: family number; PT#: patient number; NR: not-reported; AL: axial length; BCVA: best-corrected visual acuity; ERG: electroretinogram; F: female; L: left eye; M: male; mm: millimeters; NA: not available; NL: normal limit; NR: non-recordable; R: right eye; SN: subnormal; “/”: no mutation identified; + : presence; −: absence; MCOP: isolated posterior microphthalmia; MCOP/RP: posterior microphthalmia with RP; NNO: isolated nanophthalmos; NNO/RP: nanophthalmos with RP; retinitis pigmentosa: RP.

### Homozygosity mapping

Homozygosity mapping using genome-wide single-nucleotide polymorphisms (SNP) arrays was performed in three patients originating from a self-reported consanguineous marriage, using HumanCytoSNP-12 BeadChips (Illumina, San Diego, CA). Homozygous regions (>1 Mb) were identified using PLINK^28^ software integrated in ViVar^29^. Resulting homozygous regions were ranked according to their length and number of SNPs, as described^30^.

### Mutation screening by Sanger sequencing and by whole genome sequencing

Primers for PCR amplification of the coding region and splice site junctions of *MFRP* and *PRSS56* were designed (available upon request). Sanger sequencing was performed according to the manufacturer’s instructions (BigDyeTerminator v3.1 Cycle Sequencing Kit, ABI 3730XL genetic analyzer, Thermo Fisher Scientific, Waltham, MA).

One family (F7.GC20271) was included in a whole genome sequencing (WGS) study. Genome enrichment was performed using the Illumina TruSeq DNA PCR-Free Sample preparation kit (Illumina, Inc.), followed by sequencing on an Illumina HiSeq 2500 with a minimum coverage of 15x for approximately 95% of the genome. The Isaac Genome Alignment Software (version 01.14; Illumina, Inc.) was used for reads mapping against the Genome Reference Consortium human genome build 37 (GRCh37)^31^. Standard variant filtering was performed as previously described^32,33^. Structural variant (SV) assessment was done by interrogation of copy number variation (CNV), SV calls and visual inspection of the individual split and chimeric reads (Integrated Genome Viewer, IGV) across the breakpoints as described by Carss *et al*.^32^. A genomic rearrangement found in *MFRP* (GRCh37 [hg19] chr11:119, 217, 130_119, 223, 310delinsACCACTA, NM_031433.3) was confirmed using a junction PCR followed by Sanger sequencing of the junction product and by characterization of the breakpoint junctions.

### Variant interpretation

Variant classification was performed following ACMG guidelines^34^. Using Alamut Visual (v. 2.7) (Interactive Biosoftware, Rouen, France), following *in silico* prediction tools were used: Align GVGD, Sorting Intolerant From Tolerant [SIFT], MutationTaster, and PolyPhen-2, Grantham score calculation, conservation. Several genomic databases including dbSNP build 145 (http://www.ncbi.nlm.nih.gov/SNP/) and gnomAD (http://gnomad.broadinstitute.org) were used to assess variant frequencies in a general population. Segregation analysis was performed in all available family members. Mutation nomenclature uses numbering with the A of the initiation codon ATG as +1 (http://varnomen.hgvs.org/) based on the following RefSeqs: NM_031433.3 (*MFRP*) and NM_001195129.1 (*PRSS56*).

## Results

### Novel and known variants in *MFRP* and *PRSS56*

Homozygosity mapping in three families with a reported consanguineous background revealed the presence of *MFRP* in homozygous regions of 10.2 Mb and 6.2 Mb in two families (F1 and F2) respectively, while *PRSS56* was found in a homozygous region of 6.5 Mb in the third family (F18) (Fig. S1). Subsequent testing of the *MFRP* and *PRSS56* genes revealed three distinct homozygous mutations in these families, two of which are novel (Table 2). Direct testing of *MFRP* and *PRSS56* in 17 families revealed 11 additional distinct mutations in *MFRP* and *PRSS56*, five of which are novel (Table 2).

**Table 2:** Variant assessment of the identified *MFRP* and *PRSS56* mutations in 21 unrelated families with NNO or MCOP.

FAM. PID	Diagnosis	Gene	cDNA	Protein	Geno-type	Exon	Segregation	Grantham distance	SIFT	PolyPhen-2	GVGD	Mutation Taster	gnomAD (Total population frequency)	ACMG Classifi-cation	Reference
F1. B03271	NNO/RP	*MFRP*	c.1090_1094del	p.(Thr364Glnfs*26)	HOM	9	NP	**/**	**/**	**/**	**/**	**/**	0.0004102% (0 HOM)	Class 4	**This study**
F2. B14785	MCOP	*MFRP*	c.498del	p.(Asn167Thrfs*25)	HOM	5	Yes	**/**	**/**	**/**	**/**	**/**	0.0004024% (0 HOM)	Class 5	^20,38,44,45,52^
F3. GC19623	MCOP	*MFRP*	c.1549C > T	p.(Arg517Trp)	HOM	13	NP	101	D	Prob. dam.	C25	Dis. caus.	0.002022% (0 HOM)	Class 4	^41^
F4. GC19691	MCOP	*MFRP*	c.491_492insT 2nd variant unknown	p.(Asn167Glnfs*34)	HTZ	5	NP	**/**	**/**	**/**	**/**	**/**	0.005723% (0 HOM)	Class 5	^36^
F5. B09352	NNO/ RP	*MFRP*	c.498dup	p.(Asn167Glnfs*34)	HOM	5	NP	**/**	**/**	**/**	**/**	**/**	0.005723% (0 HOM)	Class 5	^8,36,41^
F6. GC18886	MCOP/ RP	*MFRP*	c.1231T > C	p.(Tyr411His)	HOM	10	NP	83	**D**	Prob. dam.	C0	Dis. caus.	Absent	Class 3	**This study**
F7. GC20271	MCOP/ RP	*MFRP*	c.955C > T	p.(Gln319*)	HTZ	8	**/**	**/**	**/**	**/**	**/**	**/**	Absent	Class 5	**This study**
			c.6087_54 +40delinsTAGTGGT	none	HTZ	5^′^UTR & 1	**/**	**/**	**/**	**/**	**/**	**/**	Absent	**?**	**This study**
F8. B10315	NNO/ RP	*MFRP*	c.498del	p.(Asn167Thrfs*25)	HOM	5	NP	**/**	**/**	**/**	**/**	**/**	0.0004024% (0 HOM)	Class 5	^36,39,42,48^
F9. B16796	MCOP	*MFRP*	c.1090_1094del	p.(Thr364Glnfs*26)	HOM	9	Yes	**/**	**/**	**/**	**/**	**/**	0.0004102% (0 HOM)	Class 5	**This study**
F10. B08047	NNO	*MFRP*	c.498del	p.(Asn167Thrfs*25)	HOM	5	Yes	**/**	**/**	**/**	**/**	**/**	0.0004024% (0 HOM)	Class 5	^36,39,42,48^
F11. GC20258	MCOP	*PRSS56*	c.833dup	p.(Val279Argfs*2)	HTZ	7	NP	**/**	**/**	**/**	**/**	**/**	Absent	Class 5	^49^
			c.1571del	p.(Val525Cysfs*55)	HTZ	13	NP	**/**	**/**	**/**	**/**	**/**	Absent	Class 5	**This study**
F12. GC18588	MCOP	*PRSS56*	c.1066dupC	p.(Gln356Profs*152)	HOM	9	NP	**/**	**/**	**/**	**/**	**/**	Absent	Class 5	^17,51,53^
F13. GC16899	MCOP	*PRSS56*	c.320G > A	p.(Gly107Glu)	HOM	4	NP	98	D	Poss. dam.	C25	Dis. caus.	0.009375% (0 HOM)	Class 3	**This study**
F14. GC19721	MCOP	*PRSS56*	c.1555G > C	p.(Gly519Arg)	HOM	13	NP	125	D	Prob. dam.	C0	Dis. caus.	Absent	Class 4	^53^
F15. RXK4624916	MCOP/ RP	*PRSS56*			HOM		Yes								
F15. RXK3297132	MCOP/ RP				HOM										
F15. RXK33447157	MCOP				HOM										
F15. RXK3113374	MCOP				HOM										
F15. RXK4633376	MCOP				HOM										
F16. RXK4941281	MCOP	*PRSS56*			HOM		Yes								
F17. RXK4985235	MCOP	*PRSS56*			HOM		Yes								
F18. B05802	MCOP	*PRSS56*	c.766T > C	p.(Cys256Arg)	HOM	7	Yes	180	D	Prob. dam.	C0	Dis. caus.	Absent	Class 4	**This study**
F19. B01921	NNO	*PRSS56*	c.766T > C	p.(Cys256Arg)	HOM		Yes								
F20. B03421	NNO	*/*	/	/	/	/	/	**/**	**/**	**/**	**/**	**/**	**/**	**/**	/
F21. B07457	NNO	*/*	/	/	/	/	/	**/**	**/**	**/**	**/**	**/**	**/**	**/**	/

Abbreviations used: FAM. PID: Family. Patient ID; HOM: homozygous; HTZ: heterozygous; NNO: isolated nanophthalmos; MCOP: isolated posterior microphthalmia; RP: retinitis pigmentosa; MCOP/RP: posterior microphthalmia with RP; NNO/RP: nanophthalmos with RP; /: no mutation identified; NP: not performed; D: deleterious; Prob. Dam.: probably damaging; Poss. Dam.: possibly damaging; Dis. Caus.: disease causing; Class 3: uncertain significance; Class 4: likely pathogenic; Class 5: pathogenic.

**Figure 1:**
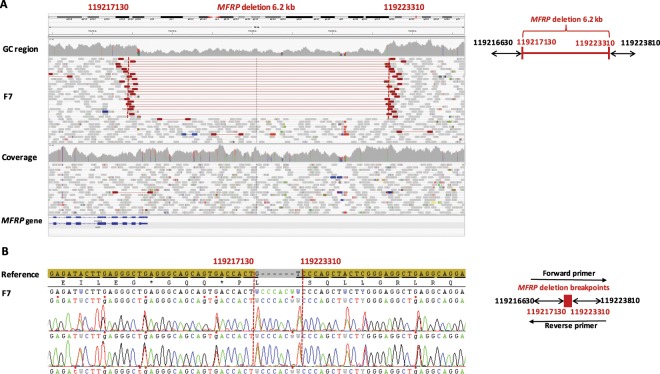
Heterozygous copy number variation implicating *MFRP* found by whole genome sequencing. The affected patient in family 7 (F7) with MCOP and RP-like changes carries a partial *MFRP* deletion. (**A**) Left panel: IGV plot generated by whole genome sequencing, showing a heterozygous deletion of 6.2 kilobases (kb) and an insertion of 7 base pairs (bps) (GRCh37 [hg19] chr11:119, 217, 130_119, 223, 310delinsACCACTA, NM_031433.3 *MFRP*: c.−6087_54 +40 delinsTAGTGGT; p.?). Highlighted red read pairs have an unusually large insert size suggestive of a large deletion, and the read depth is reduced across the heterozygous deletion. The deletion spans exon 1 of *MFRP* probably abolishing transcription. Right panel: schematic representation of the deletion. (**B**) The deletion was confirmed by junction PCR and Sanger sequencing. The arrows in the schematic left panel represent the positions of the primers.

WGS in one family (F7) revealed two novel heterozygous mutations in *MFRP*: a coding nonsense variant c.955C > T p.(Gln319*) and genomic rearrangement consisting of a deletion of 6.2 kb and an insertion of 7 nucleotides c.−6087_54 +40delinsTAGTGGT p.(?). This genomic rearrangement encompasses the 5′UTR and the coding part of exon 1 and is predicted to abolish the transcription initiation site (Fig. 1A). The breakpoints of this deletion were characterized by a junction PCR followed by sequencing (Fig. 1B). An assessment of the breakpoints at the nucleotide level showed the insertion of seven base pairs (TAGTGGT), representing a potential information scar. As no microhomology was detected at the breakpoints and only one breakpoint overlapped with an *Alu* repeat, non-allelic homologous recombination (NAHR) and microhomology-based mechanisms are unlikely. Altogether, non-homologous end joining (NHEJ) is the most likely mechanism underlying this rearrangement^35–37^.

Overall, genetic defects were found in 19 of the 21 families (90.5%): eight distinct *MFRP* mutations in ten families (10/21, 47.5%) and six distinct *PRSS56* mutations in nine families (9/21, 42.9%). Biallelic mutations were found in 18 families, while a monoallelic *MFRP* mutation was found in one family, with an undiscovered second mutation. With mutations neither in *MFRP* nor *PRSS56*, two Belgian families (2/21, 9.5%) remained molecularly unaccounted for. A summary of all variants identified, their *in silico* assessment and ACMG variant classification can be found in Table 2. Flowchart of the molecular workflow and outcomes is provided in Fig. S2.

### Phenotypic characteristics

Twenty-five patients from 21 unrelated families were investigated, six with a diagnosis of NNO and 19 with MCOP. The age of diagnosis varied from 3–75 years. Overall, all eyes had an axial length of <18 mm and hyperopia of >8 diopters with crowded discs and foveal hypoplasia. The clinical features of all studied individuals are summarized in Table 1. Based on the genotypes found, the families were divided into two genetic subtypes: a *MFRP-* and *PRSS56-*associated group.

**Figure 2:**
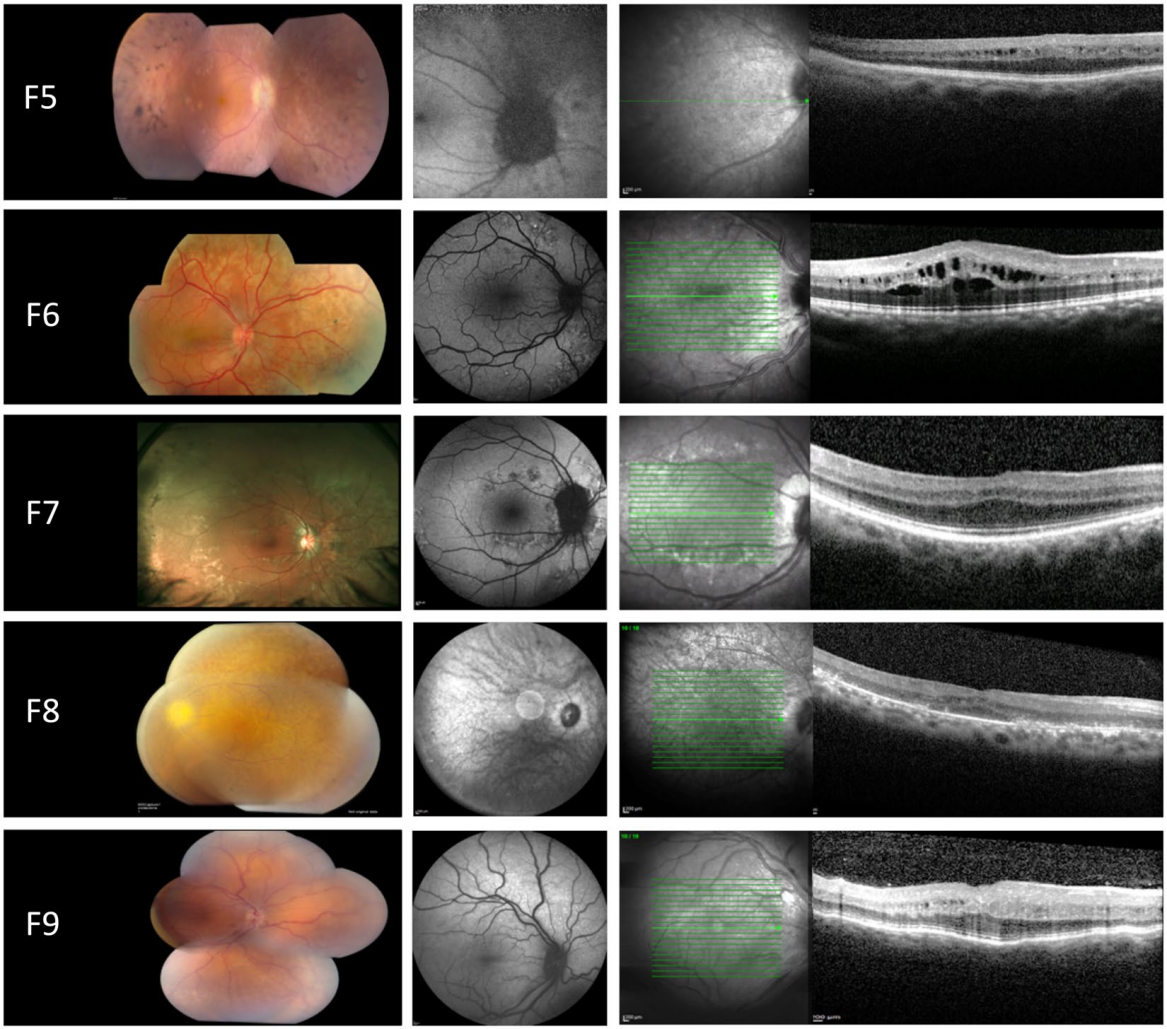
Retinal imaging from the right eye of patients with NNO or MCOP due to mutations in *MFRP*. Left panel: color fundus. Middle panel: fundus autofluorescence imaging (FAF). Right panel: optical coherence tomography (OCT). F5: color fundoscopy showing crowded optic disc, mid-peripheral intraretinal hyperpigmentation with corresponding hypo-autofluorescence on FAF, foveal hypoplasia and intraretinal cystic cavities on OCT. F6: mid-peripheral hypopigmentary retinal pigment epithelium (RPE) changes with corresponding hyper- and hypo-autofluorescence on fundus autofluorescence imaging (FAF), cystic macular cavities on optical coherence tomography (OCT). F7: posterior pole and mid-peripheral hyper- and hypo-pigmentary RPE change with corresponding hyper- and hypo-autofluorescence on FAF, thickened OCT with foveal hypoplasia. F8: color fundoscopy with crowded optic disc, slight peripheral intraretinal hyperpigmentation and large posterior pole white dots corresponding with hyper- and hypo-autofluorescence on FAF imaging, foveal hypoplasia and cystic macular cavities on OCT. F9: color fundoscopy showing crowded optic disc and normal autofluorescence on FAF imaging, thickened OCT with foveal hypoplasia and occasional intraretinal cyst.

*MFRP group*. All affected individuals had reduced BCVA ranging from 0.1 logMAR to light perception. All had crowded discs and loss of normal foveal architecture. In 3/10 (30%) of the patients, there was evidence of papillomacular folds and intraretinal cysts. Moreover, peripheral retinal pigmentary changes were observed in 5/10 (50%) patients, ranging from mild RPE hypopigmentation to extensive reticular hypopigmentation and occasional hyperpigmented lesions. ERG was performed on 8/10 (80%) of *MFRP*-mutated patients, showing subnormal ERG responses for rod and cones in 6/8 (75%) of the patients P1, P2, P4, P7, P8 and P9), a non-recordable ERG in 1/8 (12.5%) (P5) and normal ERG responses in 1/8 (12.5%) (P3). Representative retinal imaging of these individuals is shown in Fig. 2.

**Figure 3:**
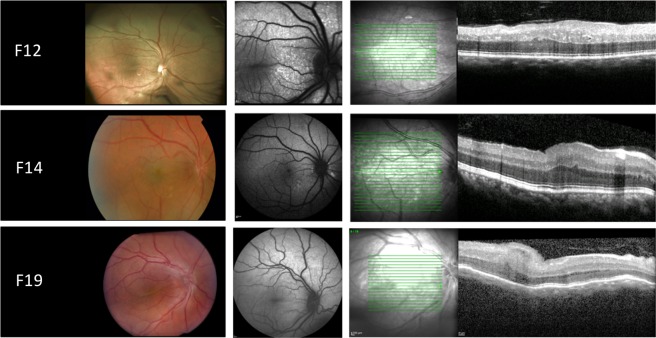
Retinal imaging from the right eye of patients with NNO or MCOP due to mutations in *PRSS56*. Left panel: color fundus. Middle panel: fundus autofluorescence imaging (FAF). Right panel: optical coherence tomography (OCT). F12: papillomacular fold with diffuse white dots in the posterior pole and to a lesser extent throughout retina, increased autofluorescence at sites of white dots, thickened OCT with occasional intraretinal cyst. F14: papillomacular fold, large posterior pole white dots that have increased autofluorescence, thickened OCT. F19: color fundoscopy showing crowded optic disc and prominent papillomacular fold, normal autofluorescence on FAF imaging, thickened OCT with foveal hypoplasia and papillomacular fold.

*PRSS56 group*. All affected individuals had reduced BCVA ranging from 0.1 to 0.8 logMAR. Crowded discs, loss of normal foveal architecture and papillomacular folds were observed in 7/13 (53.8%) patients. Peripheral retinal pigmentary changes were only found in two affected siblings (P15 and P16) (2/13, 15.4%). ERG was performed on 9/13 (69.2%) patients, showing subnormal ERG recordings in 5/13 (71.4%) (P12, P13, P14, P17 and P18), whereas normal responses were found in 4/13 (30.8%) (P11, P16 and P19 and P22). Representative retinal imaging of these individuals is shown in Fig. 3.

## Discussion

This study characterizes 21 unrelated families with NNO (n = 6) or MCOP (n = 19). Fourteen distinct *MFRP* and *PRSS56* variants were identified in the majority of the studied families (19/21, 90.5%). Eight different *MFRP* variants were found in 10/21 (47.6%) of the families, five of which are novel. Biallelic pathogenic variants were found in 9/10 families, supporting autosomal recessive inheritance. In line with the previously reported 19 distinct *MFRP* variants, mainly causing the introduction of a premature termination codon^20,38–44^, frameshift and nonsense variants represent the majority of the *MFRP* mutation spectrum of this study. Some of these variants were found to be recurrent: a novel mutation c.1090_1094del p.(Thr364Glnfs*26) in two unrelated Belgian families (F1 and F9) and a known mutation c.498del p.(Asn167Thrfs*25) in two Moroccan (F2, F10) and one Italian family (F8). The latter variant was previously reported amongst other consanguineous and non-consanguineous families of Spanish, Palestinian, Mexican and Japanese origin^9,22,38,42,45,46^. Interestingly, the previously reported reciprocal duplication c.498dup p.(Asn167Glnfs*34)^8,43^, which might point to a mutational hotspot, was found in a Dagestanian family (F5) in our cohort.

In one individual with MCOP (F4) a monoallelic *MFRP* variant was found c.491_492insT p.(Asn167Glnfs*34)^43^, with an as yet unidentified second mutation. A structural variant including a copy number variant (CNV), deep intronic mutation of regulatory mutation are possible underlying causes to be explored further. Indeed, this study provided evidence for the occurrence of CNVs affecting *MFRP* with the identification of the 6.2 kb deletion c.6087_54 +40delinsTAGTGGT.

To date, nine distinct *PRSS56* pathogenic variants have been described, mostly frameshift and nonsense mutations^17,24,47,48^. In our cohort, we detected six different *PRSS56* mutations in 9/21 (42.8%) families, some of which are known as recurrent mutations. Specifically, the previously reported missense variant c.1555G > A p.(Gly519Arg), which was found in a Saudi Arabian family^41,48^, was identified in four unrelated families of Pakistani origin in this study (F14, F15, F16 and F17), suggestive of a founder mutation in this population. Another recurrent mutation is c.1066dup p.(Gln356Profs*152) found in a Pakistani family (F12), which was previously reported in six Tunisian and five Saudi Arabian families^17,48,49^. Furthermore, two novel missense mutations were found in the *PRSS56* group in patients with isolated MCOP (F13, F18) and NNO (F19).

Overall, isolated cases with NNO or MCOP were found in 13/19 (68.4%) of the families with a molecular diagnosis, mostly in the *PRSS56*-associated group (11/13, 84.6%). Complex cases with retinal involvement (RP features or ERG changes) were found in 6/19 (31.6%) of the families with a molecular diagnosis, mostly due to *MFRP* mutations (5/6, 83.3%). The retinal involvement in the *MFRP*-associated group is in line with previous phenotypic studies^39^ and is in agreement with its expression pattern in human, mouse and zebrafish eyes including neural and pigmentary retina^3,20,21^ and its role in photoreceptor outer segment maintenance^50^. An additional explanation could be the fact that *MFRP* and a gene implicated in late-onset retinal dystrophy, *C1QTNF5* (encoding C1q and tumor necrosis factor related protein 5) are both expressed as a bicistronic transcript and found to co-localize to the same tissues with a clear functional relationship in the retina^51,52^.

The presence of papillomacular folds was found to be more frequent in MCOP (12/19, 63.2%) than in NNO (3/6, 50%). It has been proposed that these papillomacular folds result from the disparity between the retinal and scleral growth which seems to be more prominent in MCOP cases, although no clear genotype-phenotype correlations have been established yet^16,21,48^.

Finally, no clear genotype-phenotype correlations could be established in our studied cohort. For instance in the *MFRP* group both F2 and F3, having a ‘null’ allele and a missense variant respectively, displayed posterior microphthalmos without RP-like changes. On the other hand, clinical heterogeneity was observed in the *PRSS56* group, illustrated by a missense variant c.766T > C, p.(Cys256Arg) identified in F18 with a clinical diagnosis of MCOP and in F19 with a diagnosis of NNO.

Finally, a definite genetic diagnosis opens up opportunities for gene-based therapies in *MFRP*-associated retinal disease. Indeed, studies in two mouse models have demonstrated that *MFRP*-retinopathy is a potential target for gene-based therapy: *Mfrp*^*rd6*^*/Mfrp*^*rd6*^ described by Dinculescu *et al*.^53^ and *Mfrp KI/KI* described by Chekuri *et al*.^54^.

In conclusion, *MFRP* and *PRSS56* pathogenic variants, including the first genomic rearrangement of *MFRP*, were found in the majority (19/21, 90.5%) of the studied families, displaying a large phenotypic variability. No mutations were found in two Belgian families with NNO, leaving the possibility to identify underlying mutations in other NNO genes, or to uncover (a) novel NNO gene(s). Overall, this study expands the phenotypic and molecular spectrum of *MFRP*- and *PRSS56*-associated autosomal recessive NNO and MCOP in the largest cohort reported to date.

## Supplementary information

Supplementary figures.
