# Supplementary material for: The majority of autosomal recessive nanophthalmos and posterior microphthalmia can be attributed to biallelic sequence and structural variants in MFRP and PRSS56
Source: Sci Rep. 2020 Jan 28;10:1289. doi: 10.1038/s41598-019-57338-2 (PMC6987234; doi:10.1038/s41598-019-57338-2)

SUPPLEMENTAL FIGURES

The majority of autosomal recessive nanophthalmos and posterior microphthalmia can be attributed to biallelic sequence and structural variants in *MFRP* and *PRSS56*

Basamat Almoallem, MD, PhD[1,2]; Gavin Arno, PhD[3,4]; Julie De Zaeytijd, MD[5]; Hannah Verdin, PhD[1], Irina Balikova, MD, PhD[5]; Ingele Casteels, MD, PhD[6], Thomy de Ravel, PhD[7]; Sarah Hull, MD[3,4]; Martina Suzani, MD[3,4]; Anne Destrée, MD[8]; Michelle Peng, MD[9]; Denise Williams, FRCP[10]; John R. Ainsworth, MD[10]; Andrew R. Webster, MD, PhD[3,4]; Bart P. Leroy, MD, PhD[1,5,11]; Anthony T. Moore, MD, PhD[3,4,9]; Elfride De Baere, MD, PhD[1].

1. Center for Medical Genetics, Ghent University and Ghent University Hospital, Ghent, Belgium; 2. Department of Ophthalmology, King Abdul-Aziz University Hospital, College of Medicine, King Saud University, Riyadh, Saudi Arabia; 3. Institute of Ophthalmology, University College London, United Kingdom; 4. Department of Ophthalmology, Moorfields Eye Hospital, London, United Kingdom; 5. Department of Ophthalmology, Ghent University and Ghent University Hospital, Belgium; 6. Department of Ophthalmology, Leuven University Hospitals, Belgium; 7. Center for Human Genetics, University Hospitals of Leuven, Leuven, Belgium; 8. Department of Human Genetics, Institut de Pathologie et de Génétique (IPG), Charleroi, Belgium; 9. Department of Ophthalmology, University of California San Francisco, United States; 10. Birmingham Women's NHS Foundation Trust, Birmingham, UK; 11. Division of Ophthalmology, The Children's Hospital of Philadelphia, Philadelphia, Pennsylvania, United States.

Supplementary Figure 1. Outcome of homozygosity mapping and targeted mutation screening in three consanguineous families

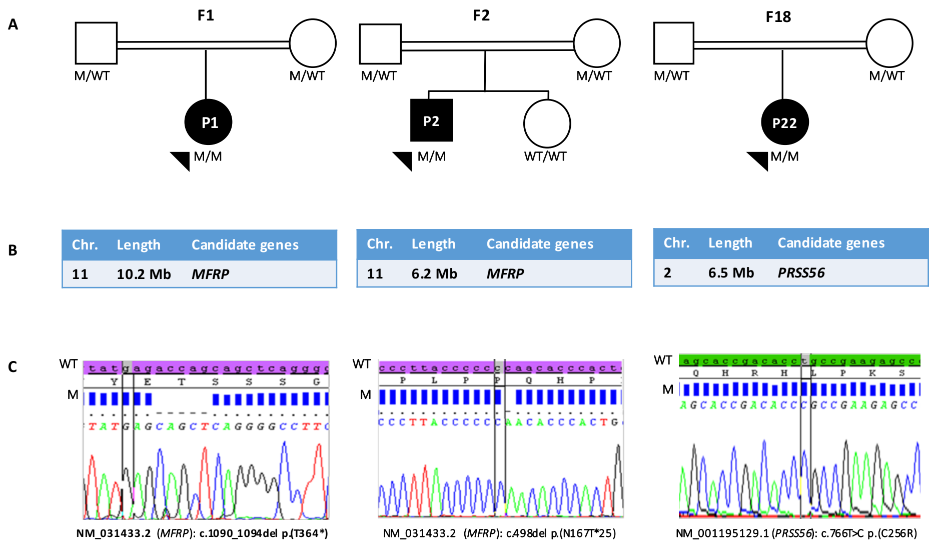

(A) Pedigrees of three consanguineous families (F1, F2 and F18). Filled symbols indicate affected individuals. Index cases are indicated by an arrow; a double line between a couple indicates consanguinity. Different genotypes are represented: homozygous mutant (M/M), heterozygous mutant (M/WT) and homozygous wild type (WT/WT). (B) Homozygosity mapping representing the size of the homozygous regions in which *MFRP* and *PRSS56* are located. (C) Sanger sequence electropherograms showing homozygous *MFRP/PRSS56* mutations.

Supplementary Figure 2. Molecular workflow in 21 unrelated families with NNO or MCOP.

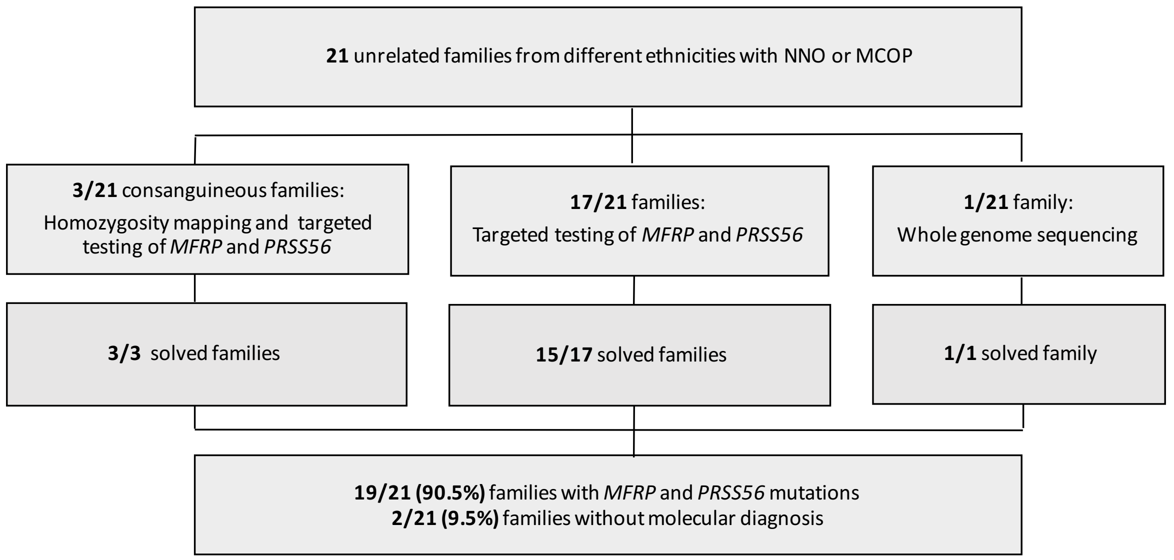

Supplement: Supplementary file 1 — Supplementary figures. [file 41598_2019_57338_MOESM1_ESM.pdf]
